# The Role of T Helper (T_H_)17 Cells as a Double-Edged Sword in the Interplay of Infection and Autoimmunity with a Focus on Xenobiotic-Induced Immunomodulation

**DOI:** 10.1155/2013/374769

**Published:** 2013-09-12

**Authors:** Nasr Y. A. Hemdan, Ahmed M. Abu El-Saad, Ulrich Sack

**Affiliations:** ^1^Department of Anesthesiology and Intensive Care Medicine, SG Sepsis Research, Center for Sepsis Control and Care, Jena University Hospital, 07740 Jena, Germany; ^2^Department of Zoology, Faculty of Science, University of Alexandria, Moharram Bey, Alexandria 21511, Egypt; ^3^Institute of Clinical Immunology, Medical Faculty, University of Leipzig, Johannisallee 30, 04103 Leipzig, Germany; ^4^University of Leipzig, Translational Centre for Regenerative Medicine, Leipzig, Germany

## Abstract

Extensive research in recent years suggests that exposure to xenobiotic stimuli plays a critical role in autoimmunity induction and severity and that the resulting response would be exacerbated in individuals with an infection-aroused immune system. In this context, heavy metals constitute a prominent category of xenobiotic substances, known to alter divergent immune cell responses in accidentally and occupationally exposed individuals, thereby increasing the susceptibility to autoimmunity and cancer, especially when accompanied by inflammation-triggered persistent sensitization. This perception is learned from experimental models of infection and epidemiologic studies and clearly underscores the interplay of exposure to such immunomodulatory elements with pre- or postexposure infectious events. Further, the T_H_17 cell subset, known to be associated with a growing list of autoimmune manifestations, may be the “superstar” at the interface of xenobiotic exposure and autoimmunity. In this review, the most recently established links to this nomination are short-listed to create a framework to better understand new insights into T_H_17's contributions to autoimmunity.

## 1. Introduction

Long-term exposure to xenobiotic substances induces hyperactivity of the immune system, thereby increasing the incidence of autoimmune diseases (AD), especially in infection-aroused systems. Circumstances dating back to earlier exposure, as in case of heavy-metal industry workers or current exposure as in individuals harboring amalgam teeth filling, favor incidence of inflammatory processes and most likely AD [1–4]. Exposure to infectious agents leads to the induction of various cellular pathways essential to the microbe's infectivity, survival, and virulence, thus making it difficult for the microbe to go undetected by the host's immune system [5]. Upon pathogen recognition, production of a proinflammatory response, primarily by macrophages, NK and NKT cells, is the subsequent event in the early phase of the infection [5, 6]. Further, the coordination between innate and adaptive immune defense systems ensures a successful eradication of pathogens, and such developed cytokine milieu determines the induction of a specific T-cell-mediated response that is critical for an effective and complete pathogen clearance. However, whether the induction of a strong host inflammation constitutes an adaptive advantage to the host or pathogen remains debated. Indeed, many disorders, including AD [7] and cancer [8], are associated with and maintained by chronic inflammation; for review, see [9, 10]. The association of cancer incidence with exposure to heavy metals, such as cadmium [11], or following attainment of chronic inflammation, as in case of colitis-associated cancer, has been widely anticipated. 

Research on T_H_17 cells has suggested a crucial role in autoimmunity. Despite developing autoimmune signs in the absence of detectable IL-17 levels, as in case of choriomeningitis-virus-induced model of type 1 diabetes [12], a key role of T_H_17 cells and their related molecules was underscored in many previously assigned “T_H_1-mediated” AD including rheumatoid arthritis (RA), psoriasis, systemic lupus erythematosus (SLE), and multiple sclerosis (MS), as well as, the experimental autoimmune encephalomyelitis—EAE [7, 9, 13–15]. Variations in disease susceptibility or outcome may be a result of co-exposure to one or multiple xenobiotic substances or infectious pathogens, so that a xenobiotic-induced polarized immune response triggers the development of AD in genetically predisposed individuals [1, 2, 4, 16–19]. The IL-17 response, while constituting a protective arm defending the body against various infections, also functions as a double-edged sword constituting a risk factor that mediates the development and/or induction of AD, mostly manifested following pathogenic and xenobiotic-induced chronic inflammation; it then acts as a double-edged sword, constituting a risk factor that mediates the development and/or induction of AD, mostly manifested following pathogenic and xenobiotic-induced chronic inflammation. In the next sections, we revisit our view on the T_H_17 cells' role in autoimmunity [9] and provide a brief description of the double-sided role of T_H_17 cells and their related molecules IL-17, IL-21, and IL-22 and their participation at the initiation/induction of autoimmunity as a consequence of xenobiotic exposure.

## 2. T_H_17 Cells and Their Associated Molecules Link Infection to Autoimmunity

**Figure 1:**
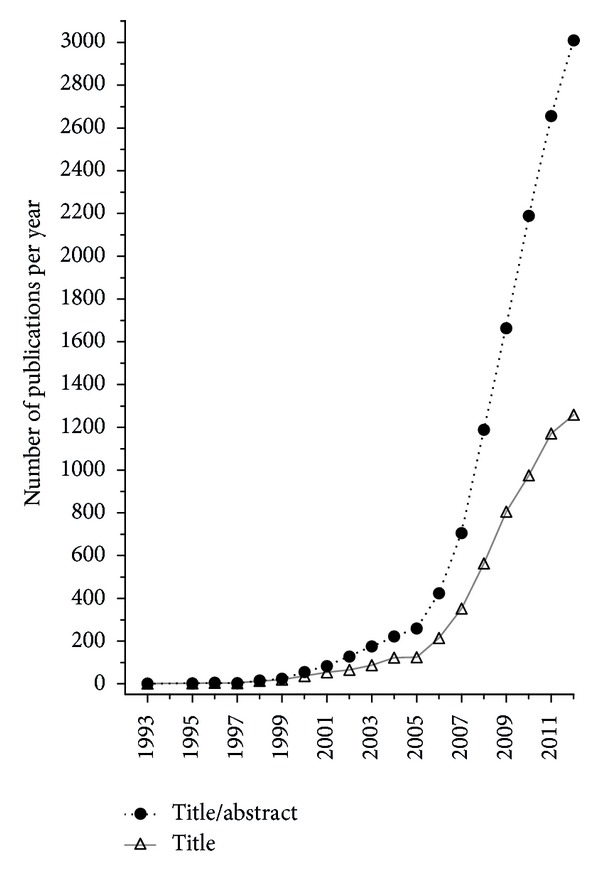
Frequencies of IL-17-producing T helper (T_H_)17 cells and their related molecules as appeared in title or title/abstract of PubMed publications. The “keywords” {T_H_17} OR {IL-17∖*} OR {IL-21} OR {IL-22} OR {IL-23∖*} OR {CTLA-8} OR {CCL20} OR {ROR*} and their synonyms combined with “publication date” {1993} through {2011} were given as search parameters.

**Figure 2:**
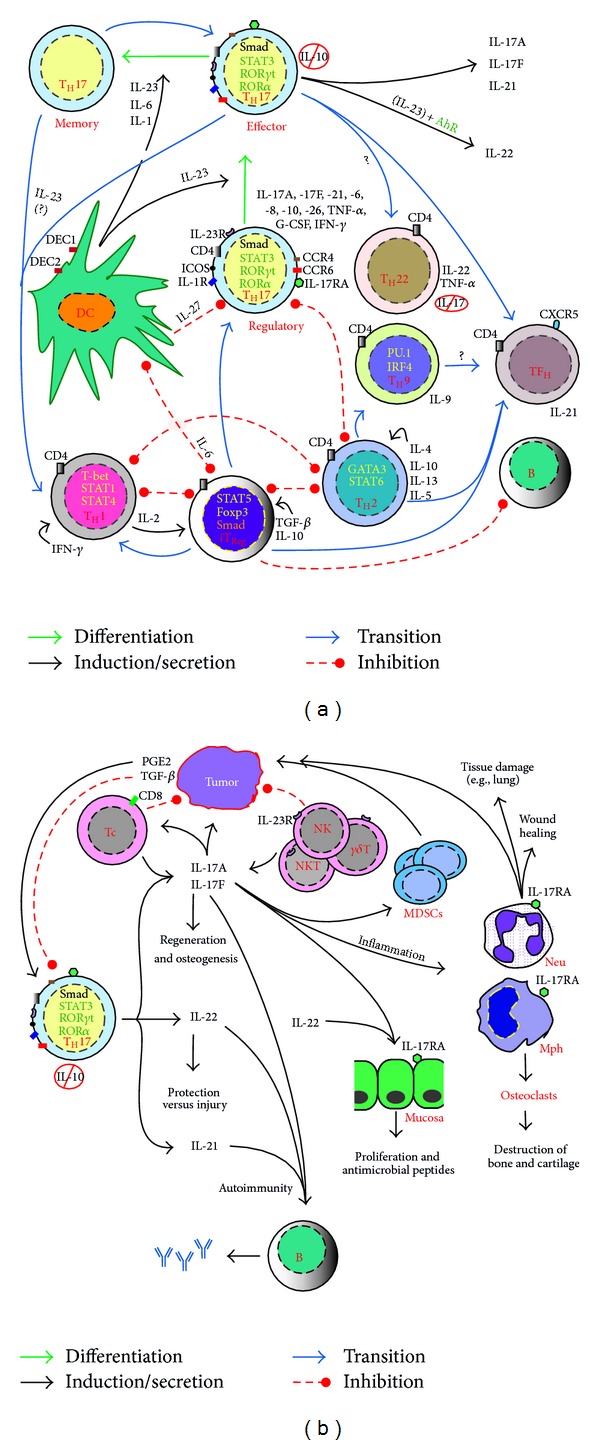
Differentiation and commitment of IL-17-producing T helper (T_H_)17 cells in the midpoint of other coacting cells in favor of and against the harboring individual. Upon antigen recognition, presentation and cosignaling, naïve (T_H_0) cells differentiate in the presence of distinct cytokine milieu into effecter (T_H_1, T_H_2, T_H_9, T_H_17, T_H_22, and TFH) and T regulatory (T_Reg_) cells. Signaling cytokine and other molecules activate lineage-unique transcription factors that ultimately mediate cell differentiation and maturation. Whereas activation of STAT1 induces T-bet expression, STAT6 signaling upregulates GATA3 expression; both cell lineages reciprocally regulate each other and regulate the generation of T_H_17 cells through their hallmark effecter cytokines, IFN-*γ* and IL-4, respectively, though IFN-*γ* is produced by T_H_17 cells in some disease settings and in response to certain infections. Differentiation of T_H_17 necessitates costimulatory signals of CD28 and ICOS (the last is not mandatory) and the absence of T_H_1 and T_H_2 cytokines (IL-12/IFN- and IL-4) and their master transcription factors; a task taken over by TGF-*β* is to constrain T_H_1 and T_H_2 during T_H_17 differentiation program. T_H_17 and T_Reg_ are likely descendants of the same ancestor cell lineage, which differentiates in presence of low levels of TGF-*β* and IL-6/IL-21 or other proinflammatory cytokines (IL-1, TNF-*α*, and IL-18) into T_H_17 cells; TGF-*β* signals through Smad2 protein pathway and is indispensable for induction of expression ROR*γ*t. High levels of TGF-*β* alone induce Foxp3 expression and hence T_Reg_ cell differentiation. Following their final commitment and upregulation of IL-23R, T_H_17 cells require IL-23 signaling that is crucial for their survival and effecter functions including production of IL-22, as well as, cell plasticity including later production of IFN-*γ*. T_H_22 may differentiate from T_H_0 cells or through a local commitment of T_H_17 cells homed in the epidermis. Contributions of T_H_17 cells entail activations of aryl hydrocarbon receptor (AhR) signaling and production of IL-22 upon exposure to xenobiotic substances. IL-17 and IL-17F increase production of IL-6, IL-8, prostaglandin E2 (PGE2), monocyte chemotactic protein-(MCP-) 1, and the granulocyte colony-stimulating factor (G-CSF) by various cells including macrophages, fibroblasts, keratinocytes, and epithelial and endothelial cells and ultimately promote inflammatory diseases, AD, and/or cancer. These cytokines together with IL-21 and IL-22 are also implicated in mediating protective as well as pathogenic processes in various disease settings.

T cells differentiate and expand into distinct lineages including T_H_1, T_H_2, iT_Reg_, and T_H_17 cells [9], whereas iT_Reg_ cells differentiate under subimmunogenic antigen presentation both during chronic inflammation and under normal homeostatic conditions of the gut and function to control severe chronic allergic inflammation and as a barrier to the eradication of tumors [20, 21]. T_H_17 cells derive from CD161^+^ precursors in umbilical cord blood and newborn thymus [22] and likely constitute the most prominent T cell subset at the crossroads of infection and autoimmunity. The contributions of T_H_17 cells have prompted and were the results of intensive scientific research, which is reflected by a growing list of publications in this field (Figure 1), and have in turn led to identification of T_H_17 cells' markers, as well as, their differentiation and commitment program [23].  Figure 2 demonstrates the major T cell subsets, their interaction with T_H_17 cells and the main contributions of the latter. 

Recently, several groups delivered compelling evidence of the effects of T_H_17-associated cytokines, namely IL-17, IL-21, IL-22, and IL-23, on inflammatory responses elicited by extracellular, as well as, facultative and obligate intracellular pathogens including bacteria and fungi. Exemplified contributions of IL-17 response to some infectious diseases are summarized in Table 1. 

### 2.1. T_H_17 Cells-Associated Molecules and Their Contributions to Anti-Infectious Responses

In comparison to the frequent appearance of T_H_1, the relative rarity of T_H_17 in inflamed tissues was attributed to their *RorC*-dependent expression of the oxidase IL4I1, which impairs CD3 signaling and hence constrains IL-2 production and cell proliferation [24]. As we recently reviewed, the recruitment of T_H_17 cells to inflammatory tissues accompanies the expression of the chemokine receptor CCR6, in addition to CCR4, IL-23R (involved in the survival/maturation program of T_H_17 cells) [9], and CD161 [22]. T_H_17 cells are considered as potent inflammation inducers that, in addition to production of IL-17, differentially produce IL-6, IL-2, IL-8, IL-9, TNF-*α*, IL-17F, IL-21, IL-22, IL-26, IFN-*γ*, and the chemokine CCL20 and induce activation and recruitment of other cells including neutrophils that are pivotal in inflammation and AD [9, 25]. Through their cytokine/chemokine production, T_H_17 cells act on a broad range of cell types initiating the expression of antibodies, metalloproteinases, prostaglandin E2 (PGE_2_), and antimicrobial peptides and inducing cyclooxygenase 2 activity [9, 26], constituting, thereby, a link between innate and adaptive immune responses. In addition to its role as an arm of adaptive immunity, the current perception categorizes IL-17 also as an innate cytokine, produced mainly by NK cells [27] as well as by *γδ* T cells [28–31], CD8^+^ T cells, and mast cells [9, 32]. Indeed, detecting functional T_H_17 cells and production of protective IL-17 during the early phase of the immune response [33–35] and the activation of T_H_17 that even precedes the differentiation of T_H_1 cells [33], together with the later contribution of T_H_17 [36], highlight the crucial importance of IL-17 and other T_H_17-related cytokines in the early, as well as, the late phase of infection. The upregulation of TLR1 and TLR2 and dectin 1 by IL-17-producing *γδ* T cells [37] supports this belief. Moreover, studies on nucleotide oligomerization domain knockout mice (Nod1^−/−^ and Nod2^−/−^) demonstrated that this “early” T_H_17 response was Nod1- and Nod2-dependent, and hence they have been given the name innate (i)T_H_17 cells [35]. Therefore, the new look of IL-17-producing cells comprises their contribution in building the first line of host defense, besides mediating and shaping adaptive responses required for ultimate clearance. 

Based on a wealth of experimental data, the contribution of T_H_17 cells to infection is manifold. As in the case of oral infection, the importance of IL-17 in protection against infection seems to be crucial to attain a mucosal barrier in the intestine as in case of *Salmonella* [30, 38], in mediating protection against oral brucellosis [39], promoting granulopoiesis through induction of granulocyte colony-stimulating factor G-CSF [40], and neutrophil influx through inducing neutrophil chemotactic CXCL8 (IL-8), macrophage chemotactic protein (MCP)-1, and macrophage inflammatory proteins- (MIP-) 1 and MIP-2 [9, 40, 41]. Additionally, we and others found that IL-17 activates phagocytosis and neutrophil cytotoxic activity [42]. Therefore, IL-17R^−/−^ mice revealed increased systemic dissemination of *S. typhimurium* from the gut [29]. The same strategy seems to be attained to combat extracellular pathogens such as *Klebsiella pneumoniae* [12] and fungal infections (e.g., *Candida *sp.), mainly due to defective IL-17 immunity [43, 44], mediated by eliciting production of autoantibodies (AAs) against IL-17, IL-17F, and IL-22 that contribute to chronic mucocutaneous candidiasis [45, 46]. A third arm of T_H_17 cells is built through IL-22, which is also produced by other cell types including NK22 and lymphoid tissue inducer cells [47], as well as by skin homing T_H_22 cells [48, 49]. Besides its accepted role against infection, it induces tissue repair offering protection against injury [49]. Although both IL-22 and IL-17 or IL-17F synergize to stimulate expression of human beta-defensin- (HBD-) 2, S100 calcium binding protein A9 (S100A9) and enhanced the expression of S100A7 and S100A8 [9], IL-22, rather than IL-17, seems to contribute more to the epidermal and mucosal immunity [47, 49]. It synergizes with TNF-*α* to induce secretion of initial complement factors C1r and C1s, antimicrobial peptides S100A7 and HBD-2, and antimicrobial chemokines CXCL-9/-10/-11 in primary human keratinocytes [50]. In a three-dimensional skin infection model, stimulation of keratinocytes with T_H_22 supernatants or by adding IL-22 plus TNF-*α* effectively inhibited *C. albicans* growth and maintained epithelial survival, and the combinatorial stimulation of keratinocytes with IL-22 plus TNF-*α* most effectively conserved the integrity of the epidermal barrier as compared with IFN-*γ*, IL-17, IL-22, or TNF-*α* alone [50]. IL-22 also functions to induce an acute phase systemic response that extends beyond IL-22R-expressing cells and revealed diverse significant impact on coagulation and cellular constituents of blood, in addition to induction of thymic atrophy, body weight loss, and renal proximal tubule metabolic activity and biochemical changes in the liver, including induction of fibrinogen, CXCL1, and serum amyloid A [51]. Besides its contribution to protection against bacterial infection [50, 52], IL-22 plays an important role in protection against viral infection, for example, hepatitis B virus [53]. On the other side, IL-22 is implicated in the induction of IBD [54] and AD such as experimental autoimmune myocarditis [9] and psoriatic disease through the induction of keratinocyte proliferation and cytokine and chemokine release [7]. This reflects the dark side of the T_H_17 story; that is, a promoted T_H_17 response may reflect a current or predict incidence of AD. 

### 2.2. T_H_1-T_H_17 Cells Interaction during Infection

Although a protective role against intracellular bacteria such as* Listeria monocytogenes* [55] or *S. typhimurium *(N. Y. A. Hemdan and A. M. Abu El-Saad, unpublished data) may be attributed to T_H_17 response, this may be rather compensatory to a defective IL-12/IFN-*γ* axis as previously demonstrated by N. N. Orgun et al. [56], or a complementary function to indirectly induce type 1 response mediated by APCs endowing thereby a protection against infection, as in case of the obligate intracellular bacteria *Chlamydia muridarum* [57]. In case of infection with *S. typhimurium* and *C. muridarum*, neutralizing IL-17 significantly reduced pathogen-specific T_H_1 but promoted higher T_H_2 responses. DCs isolated from IL-17-neutralized mice demonstrated lower expression of CD40, MHC II, and IL-12 production, but higher level of IL-10 compared with control mice [57]. Furthermore, neutralizing IL-17 in case of *S. typhimurium* significantly reduced phagocytosis as well as T_H_1 cytokine production (N. Y. A. Hemdan and A. M. Abu El-Saad, unpublished data). Moreover, delivery of an IL-17R antagonist that resulted in a 50% reduction in the neutrophilic infiltration in lungs following *Chlamydia* infection reversed the susceptible phenotype of C3H/HeN mice [58], indicating a key role of IL-17 in induction of neutrophil infiltration. The compromised IL-17 response in HIS (Job's syndrome) patients that contributed to higher susceptibility to *Staphylococcus aureus* infection [59] is evidenced by a recent finding that coinfection with influenza A abrogated host defense, which was rescued by overexpression of IL-23 and markedly improved bacterial clearance [52]. Influenza A was found to inhibit T_H_17 differentiation and substantially decreased IL-17, IL-22, and IL-23 production after *S. aureus* infection. Interestingly, IL-17-mediated cross-protection against secondary *L. monocytogenes* infection has been demonstrated following immunization with *Mycoplasma pulmonis* [60]. 

In addition to the function of T_H_17 cells as a substitute for a defective T_H_1 response, synergism between T_H_17 and T_H_1 cells is proposed following infection or postvaccination challenge with *Mycobacterium *sp., based on the observation that IL-17^−/−^ mice revealed a reduced IFN-*γ* production by CD4^+^ T cells and impaired granuloma formation and expression of chemokines CXCL9, CXCL10, and CXCL11 [61]. Also, an enhanced T_H_1 memory response in the lungs of vaccinated mice infected with *M. tuberculosis* was dependent upon IL-23/IL-17 axis [61]. In a model of TCR *αβ* ^−/−^ mouse [62], where adoptive transfer of either T_H_1 or T_H_17 cells restored bacterial burdens and innate immune cell infiltrates to wild-type animals level, T_H_17 transferred cells revealed plasticity within the CNS compartment with an ultimate T_H_1-like cytokine profile, and this might be the reason for restoration of a strong innate immune response against infection with pyogenic bacteria; for review on various forms of T_H_17 cell plasticity, refer to Hemdan [10]. Furthermore, the importance of T_H_17 cells in combating HIV-associated bacterial infections has been recently elucidated [63]. 

### 2.3. Interaction of T_H_17 Cells with Commensal Bacteria

Of a crucial importance is the recent clue linking the induction of T_H_17 response with gut commensal bacteria. Colonization of the small intestine of mice with a single commensal microbe, segmented filamentous bacterium (SFB), was sufficient to induce IL-17- and IL-22-producing T_H_17 cell responses in the lamina propria, and this was correlated with enhanced expression of inflammation- and antimicrobial-associated genes and increased resistance to the intestinal pathogens *Citrobacter *and *Salmonella* [35, 64]. Induction of T_H_17 cells mediated autoimmune arthritis in K/BxN mice [65], whereas when the same mice were held under germ-free conditions, autoimmune arthritis was strongly attenuated and mice revealed reductions in serum AAs titers, splenic AAs-secreting cells, germinal centers, and splenic T_H_17 cells as well as the lack of T_H_17 cells in the small intestinal lamina propria [65]. These findings suggest the role of T_H_17 cells not only in defending the gastrointestinal tract against pathogens, but also in mediating AD (Table 1). How does the immune system monitor the resident intestinal microbes and coordinate between host defense and tolerance and how do dysregulated host-microbe interactions lead to intestinal inflammation were recently discussed [66]. The increased production of IL-17 and IL-23 by PBMCs derived from patients of primary Sjogren's syndrome upon TLR2, TLR4, and TLR6 stimulation [67] highlights the link between TLR ligation and autoimmune induction in such disease settings, where the participation of T_H_17 cytokines in their pathogenesis is evident [68, 69]. 

### 2.4. T_H_17 Cells in the Bone Disease

One of the most important contributions of T_H_17 cells involves bone metabolism and bone disease. The coincidence of chronic inflammation and osteoporosis (OP) or osteoarthritis (OA) is quite anticipated and raised a debate about IL-17's contribution. Several hallmark inflammatory mediators including TNF-*α*, IL-1, IL-6, IFN-*γ*, receptor activator of NF-*κ*B (RANK), and RANK ligand (RANKL) are of crucial importance not only at the primary inflammation site, but also in bone metabolism [70, 71]. Although a protective role of IL-17 against bone loss has been described [72], induction of osteoclastogenesis by T_H_17 cells has been suggested in various inflammatory models [73]. Proinflammatory cytokines correlated with osteoclastogenic or antiosteoclastogenic manifestations in human OP and OA, for example, negative correlations of hip bone mineral density (BMD) with TNF-*α* in OA and with RANKL/RANK in OP [70]. In a mouse model of type II diabetes, whereas osteocalcin and osteoprotegerin (osteoblast-specific bone forming markers) were decreased, osteoclast-driven bone resorption markers such as IL-6 and RANK were elevated and coincided with enhanced RANKL and IL-17 expression by CD4^+^ cells; IL-17 induction was directly promoted upon leptin treatment [74]. The authors proposed that leptin and IL-6 stimulate IL-17 production and, thereby, induce RANKL-mediated osteoclastogenesis. A direct link of IL-17 to osteoclast induction was proved in cultures of PBMCs drained from patients with Crohn's disease [71]. Altogether, IL-17 may be a valuable target for controlling bone diseases, at least those accompanying chronic inflammations as in Crohn's disease or inflammatory arthritis. 

Overall, in addition to expecting counterprotective impacts of T_H_17-associated cytokines, the induction of T_H_17 response seems to be an intrinsic feature originally evolved to fight bacterial, viral, and fungal infections. However, what drives such an immune arm to react against the body's own elements, that is, the loss of tolerance, remains elusive. Upon infection, it seems to be a failure to eliminate the invader, whereby an inflammation-potent cell response is amplified, whose army calls for other inflammation competent cells that might have lost the ability to recognize the body's own MHC molecules and therefore attack the self and/or induce production of AAs. Such a modified response attained through a persistent infection constitutes an additional load against the system's strategy of pathogen clearance and the culmination of the immune response to its steady state thereafter. In other words, boosting such a potent inflammatory cell type as T_H_17 through an initial inflammation, for example, through inflammatory cytokine-mediated induction of NF-*κ*B, see next, should normally be accompanied by induction of a regulation program; otherwise autoimmunity occurs. On the basis of current understanding, we propose that the T_H_17-driven autoimmune response is manifold, attaining its incidence through (i) activating T_H_1 responses and the later conversion of T_H_17 themselves into T_H_1-like cells or double T_H_1/T_H_17 cytokine producers having the inflammatory potency of both subsets; (ii) activating B cells and their production of AAs, especially through IL-21-dominated responses; (iii) inducing inflammatory cells like macrophages and neutrophils and their recruitment through induction of chemokines, facilitating thereby tissue destruction and release of intrinsic cellular factors, which, in turn, leads to local or systemic induction of the autoimmune traits; and (iv) promoting cytotoxicity of NK and CD8^+^ cells and the conversion of the latter cells into IL-17 producers that further magnify the whole response. On the basis of current knowledge, introducing the T_H_17 efficacy as a potent inflammatory lineage, targeting IL-23/T_H_17 axis, may be a promising approach that paves the way for additive and alternative treatment of chronic inflammation and AD [75]. 

## 3. T_H_17 Cells Are Key Players in Heavy-Metal-Elicited Autoimmunity

Whereas some heavy metals (e.g., copper, selenium, iron, and zinc) are essential to maintain our metabolism, the majority of heavy metals are non-essential, for example, arsenic (As^3+^, As^4+^), cadmium (Cd^2+^), chromium (Cr^3+^, Cr^4+^), mercury (Hg^2+^), and lead (Pb^2+^), and are ranked among the most highly toxic substances. Great evidence exists that various heavy metals elicit immunomodulation increasing thereby the incidence of human AD and cancer [3, 18, 76]. It has been recently found that patients with autoimmune thyroiditis (AT) and other AD, including MS, psoriasis, SLE, and atopic eczema, showed increased lymphocyte reactivity to inorganic Hg^2+^, Ni^3+^, and other metals and that replacement of amalgam in Hg^2+^-allergic subjects resulted in improvement of health in about 70% of AT patients [3]. Furthermore, recent data implied that exposure of mice to low micromolar concentrations of Cd^2+^ and Hg^2+^ induces a robust T_H_17 response, that was also inferred by mild but significant increase of IL-17 profile in serum of individuals occupationally exposed to the same metals, as well as a robust *ex vivo *T_H_17 response (N. Y. A. Hemdan et al., unpublished data).

### 3.1. Ligation of Metal Ion with the Aryl Hydrocarbon Receptor

Like other xenobiotic stimuli [77], one mechanism so far delineated is the ligation of metal ion with the aryl hydrocarbon receptor (AhR) as in the case of Cd^2+^, As^3+^, Cr^6+^ [78, 79], and Pb^2+^ [80]. Such ligand-specific activation of the AhR was found to regulate the balance between T_H_17 and T_Reg_ cell responses [81]. Whereas AhR activation by TCDD (dioxin) induces functional T_Reg_ cells that suppress EAE, activation by 6-formylindolo[3,2-b]carbazole induces T_H_17 cells and ultimately the disease severity. AhR is expressed by T_H_17 cells, *γδ* T cells, and DCs [82, 83] and is indispensible for IL-22 production as evidenced by AhR^−/−^ mouse studies [82] and by downregulation of the AhR on RNA-mediated interference, as well as, by applying AhR agonists [48], where it substantially altered the balance of IL-22- versus IL-17-producing cells. In DCs, activation of AhR induces expression of IDO1 and IDO2 that mediate induction of T_Reg_ cells [83]. Therefore, we hypothesized that exposure to heavy metals may mediate autoimmune initiation/induction through metal ligation of AhR. Recent works of other groups and our unpublished data indicate the association of IL-22 with the appearance of autoimmune signs, as inferred in the pathogenesis of psoriasis [7]. These data raise the AhR as a sequential segment linking such potent inflammatory T_H_17 cells with the heavy-metal-induced autoimmune induction and reveal a mechanism for further differentiation of T_H_17 into T_H_22 under organ- or pathogen-specific conditions, including AhR ligation pathways. Therefore, targeting AhR may offer a possibility for differential regulation of T_H_17 cytokines and thereby reduction of autoimmune susceptibility in heavy-metal occupationally exposed individuals; however, the paradoxical effect of various AhR ligands should be considered. 

AhR-mediated immunomodulation by heavy metals may involve several mechanisms; one of which is the regulation of CYP1A1 expression, for example, in the case of Pb^2+^ [80] that coincided with increase of heme-oxygenase- (HO-) 1 mRNA level and production of reactive oxygen species (ROS). Induction of CYP1A1 expression by AhR ligation is well documented; its downstream signal mediates cellular responses probably through modifying cytokine secretion, including IL-6 [84], IL-17, and IL-22 [37, 82, 85]. Induction of oxidative stress has been reported in case of Pb^2+^ [86], Cd^2+^ [87–89], and Hg^2+^ exposure [90], inferred by reduced activity of antioxidant enzymes catalase (CAT), superoxide dismutase (SOD), and glutathione peroxidase (GPx) and reduction of glutathione (GSH). Smoking, a major source of Cd [91], also initiates ROS production accompanied by augmented cell signaling pathways implicated in the pathogenesis of AD such as psoriasis that implicates mitogen-activated protein kinase (MAPK), NF-*κ*B and Janus kinase JAK/STAT [92], and reduced antioxidants malondialdehyde and SOD [93]. Oxidative stress comprising production of free radicals such as reactive oxygen and nitrogen species is closely related to inflammation and discussed as an underlying mechanism of inflammatory diseases, accompanying activation of the leucocytes and generation of peroxynitrite, at the early stages of induction, or appearing before the incidence of various AD such as IBD [94, 95], systemic sclerosis [96], SLE [97], psoriasis [93, 98], and in cardiovascular inflammation [99]. Therefore, depletion of endogenous antioxidants such as GSH, Cu, and ZnSOD was manifested in experimental models of IBD [100], accompanied, however, by induction of HO-1. The latter catalyzes CO production, and, although it is considered as a prooxidant due to iron released from HO activity [101], it may be a compensatory response to oxidative stress and chronic inflammation [102], whose protective function has been elucidated in various AD including MS [103].

The protective role of antioxidants in combating inflammation has been clarified in various models by applying exogenous antioxidants or by manipulating expression of endogenous antioxidants. Depletion of NF-E2-related factor 2 (Nrf2), for instance, markedly enhanced susceptibility of experimental IBD [104]. Moreover, by disrupting GSH metabolism through targeting GSH peroxidase (GPx) 1 and GPx2 derived development of colitis [105], or by depleting GSH though curcumin-elicited glyoxalase 1 activity inhibition that enhanced the anti-inflammatory response as well as anticarcinogenetic potency [106], it became clear how close the metabolic stress is related to cell response and cell survival, an assumption that is confirmed by many studies [107]. Unfortunately, although the role of T_H_17 cells-related products such as IL-17 in the previous experimental settings has not been addressed, T_H_17 together with T_H_1 cells are drawn as being major players in the IBD as well as in other inflammatory concerts [108]. Therefore, the elevation of ROS levels links heavy metal exposure to induction of inflammation and cancer. Indeed, induction of T_H_17 cell response by heavy metals is likely a downstream event of hydrogen-peroxide- (H_2_O_2_-) mediated IL-6 induction, which is found to protect resident lung cells from ROS-induced injury [109]. Ye et al. [110] found that fumarates induced type II DCs as a result of initial GSH depletion followed by induction of HO-1, which interacts with AP-1 and NF-*κ*B sites of *Il23p19* promoter and inactivates STAT1 and thereby improves T_H_1- and T_H_17-mediated AD including MS and psoriasis.

### 3.2. Modification of NF-*κ*B Signaling

A key pathway through which heavy metals, amongst other xenobiotic substances, exert impacts on the immune response occurs via modifying NF-*κ*B signaling. Several distinct NF-*κ*B activation pathways are identified, including responses to various cell stresses and stimuli such as proinflammatory cytokines TNF-*α* and IL-1, bacterial products, genotoxic stimuli such as ionizing radiation and some chemotherapeutic drugs [88], in addition to exposure of various cell types to heavy metals such as Cd^2+^ [88, 111]. This may highlight NF-*κ*B activation as a trait of carcinogenicity assigned to heavy metals including Cd^2+^ [112]. The increased production of cytokines, for example, IL-6 and IL-17, in murine models and in heavy-metal-exposed individuals (Hemdan & Abul El-Saad, unpublished data), might ultimately lead to excessive induction of NF-*κ*B-mediated chronic inflammation [113]. This is consistent with the involvement of IL-17 in the differentiation of plasma cells mediated by NF-*κ*B-regulated TF Twist-1 [114]; we recall the correlation of higher IL-17 levels with the severity of various AD and the appearance of autoimmune signs accompanying exposure to heavy metals. Therefore, delicate intervention to regulate NF-*κ*B activation may help prevent chronic inflammation, simultaneous tissue cell damage, and reduce incidence of AD in individuals occupationally exposed to heavy metals or those with an accidental exposure history. 

### 3.3. Disruption of Ca^2+^ Homeostasis

A third event by which heavy metals like Cd^2+^ modify cell survival and function is modifying Ca^2+^ displacement and ultimately adherence/tight junctions, mediated by disrupting expression and translocation of E-cadherin/*β*-catenin, in a way that mimics Wnt-signaling [88, 115], providing a clue for the carcinogenicity of heavy metals. Previous studies [116] as well as our unpublished data identified some protein kinases including ROCK-II as a target of Cd^2+^- and Hg^2+^-mediated induction of IL-17, which activates NF-*κ*B through CIKS/Act1 adaptor proteins, inducing thereby chronic inflammation and cancer. 

### 3.4. Modification of Zinc Metabolism

A further important element in the interplay of heavy metals such as Cd^2+^ with immunomodulation is represented by its interaction with zinc. Zinc was found to inhibit cancer through regulating various oncogenic pathways including NF-*κ*B, AP-1, Notch-1, and PI3K/Akt, apoptosis, cytotoxicity, regulating tumor suppressors such as p53 and macrophage phagocytic activities and increased production of ROS and inflammatory cytokines TNF-*α*, IL-1*β*, IL-8, VCAM, and MCP-1 [112]. Similarly, Zn probably inhibits STAT3 activation and thereby T_H_17-mediated collagen-induced arthritis [117]. Via targeting such oncogenic pathways, Zn supplements may participate in attaining promising antitumor approaches, at least in cases where Cd^2+^ is considered to have a carcinogenic potential. 

## 4. Concluding Remarks

Ongoing research provides a preponderance of evidence that T_H_17 cells and related molecules act as double agents both in favor of but also against the harboring individual. They elicit various antimicrobial mechanisms on one hand, but, on the other hand, when dysregulated, likely triggered by xenobiotic agents including pollutants and infectious agents, initiate/promote chronic inflammatory/autoimmune manifestations. A delineation of the underlying mechanisms that culminate into hyperactivation of T_H_17 cells and the resultant production of related mediators would facilitate the development of potential therapeutic approaches to combat their deteriorating effects but simultaneously allow their benefits to act. Therefore, various research directions gave more attention in the last decade to T_H_17-cells-related molecules to help attain and evaluate valuable therapeutic strategies. Manipulating T_H_17 differentiation and function by targeting differentiation/promoting cytokines, transcription factors, or commensal-bacteria-elicited immune induction may be valuable for treating AD; however, the risk of increasing the vulnerability of attacking infections should remain in focus. Therefore, it may be of worth to apply prophylactic antibacterial and antifungal therapy in case of treating patients of AD with IL-17/-22/-23 inhibitors.

## Take-Home Messages

- T_H_17 cells and their related products act as double agents both to mediate various antimicrobial mechanisms and to initiate/promote chronic inflammatory/autoimmune manifestations.
- Simultaneous or successive exposure to xenobiotic substances and infectious agents renders the genetically susceptible individual vulnerable to autoimmune incidence via induction of inflammatory mediators.
- A compromise should be met to facilitate development of potential therapeutics aiming at targeting AD via inhibiting differentiation of T_H_17 cells and/or commitment factors for the benefit of attaining the antimicrobial response intact, for example, by applying prophylactic therapy combined with IL-17/-22/-23 inhibitors.

## Figures and Tables

**Table 1:** Mechanistic investigations on the role of T_H_17 cells and their related molecules in various infectious diseases.

Diagnosis	Role	Observations on T_H_17-associated molecules	Citations
(1) Bacterial infections			

*Bacillus subtilis *	Pathogenic/protective	Increased lung inflammation and collagen deposition; delay in bacterial clearance in IL-17R^−/−^ compared with WT counterparts	[118]
*Francisella tularensis *	Pathogenic	Intranasal inoculation induces T_H_17 response and PGE_2_ production in the lung; inhibition of PGE_2_ production increased IFN-*γ* and decreased bacteremia	[119]
*Saccharopolyspora rectivirgula *	Pathogenic	Induction of IL-17-mediated hypersensitivity pneumonitis in mice; reduced lung inflammation and fibrosis in IL-17R^−/−^ mice	[120, 121]
*Klebsiella pneumoniae, Bordetella pertussis, *& *S. pneumoniae *	Protective	Mounting of an IL-17 and IL-22 response; defects in T_H_17 response increased susceptibility	[12, 122]
*Staphylococcus aureus *	Protective	High infection incidence correlated with defect in T_H_17 response	[59]
*Listeria monocytogenes *	Protective	IL-17-mediated cross-protection following immunization with *M. pulmonis*; blockade of bacterial growth following transfer of IL-17-producing *γδ* and double negative *αβ* T cells into RAG2^−/−^ mice	[30, 60]
*Shigella flexneri *	Protective	Restriction of bacterial growth mediated by T_H_17 response	[28]
*Citrobacter and Salmonella *sp.	Protective	Innate T_H_17 response-dependent protection; protective effect of IL-17 and IL-22; decrease in phagocytic activity and increase in bacterial burden upon IL-17 neutralization and its correlation with T_H_17 response in Hg-exposed mice	[29, 35, 38] and Hemdan and Abul El-Saad, unpublished
*Mycobacterium tuberculosis and M. bovis *	Protective	IL-17^−/−^ mice reveal a reduced IFN-*γ* production by CD4^+^ T cells, impaired granuloma formation, and chemokine expression	[61]
*Mycobacterium tuberculosis *	Protective	Correlation of reduced T_H_17 responses in patients with active tuberculosis with decreased expression of IL-6R on CD4^+^ T cells	[123]
*Chlamydia *sp.	Protective	Enhanced bacterial growth and decreased mouse survival upon applying anti-IL-17 mAb	[34]
	Pathogenic	Applying IL-17RA antagonist reversed the susceptible phenotype of C3H/HeN mice	[58]
Inflammatory bowel disease—IBD (Crohn's disease and ulcerative colitis)	Pathogenic	Enhanced differentiation T_H_17 and IL-17 expression levels and NK activities in IBD	[124, 125]
	Protective	IL-22 mediated protection against IBD	[54]

(2) Protozoal infection			

*Toxoplasma gondii *	Pathogenic	IL-23-mediated IL-22 and MMP-2 upregulation in the ileum of infected mice; MMP-2 deficiency offered protection	[47]
	Protective	Increased mortality in IL-17^−/−^ mice	[126]

(3) Fungal infections			

*Candida *sp.	Protective	Involvement of IL-17, IL-17F, IL-22, and IL-23 in mediating natural defense against candidiasis	[45]
*Aspergillus fumigatus *		Induced IL-17 response mediates pathogen clearance	[127]

(4) Viral infection			

Theiler's murine encephalomyelitis virus infection	Pathogenic	Induction of antiapoptotic molecules by IL-17 and thereby promoting persistent infection; boosting lytic function of CTLs and ameliorating disease upon neutralizing IL-17; association of lower T_H_17 with higher virus-specific CD8^+^ T cell responses in resistant mouse than in susceptible strain	[128]
Respiratory syncytial virus (RSV)	Pathogenic	Elevated IL-6 and IL-17 levels in tracheal aspirate samples from severely ill infants and in infected mice; IL-17 blockade decreased the exacerbated disease via increasing RSV-specific CD8^+^ T cells, T-bet, IFN-*γ*, eomesodermin, and granzyme B	[129]
HBV	Pathogenic/protective	Distinct effects associated with heterogeneous T_H_17 populations: IL-17 with inflammation and ALT levels, IL-22 with protection of hepatocytes, and IL-21 with virus clearance	[130]
HCV	Pathogenic	Hepatitis-C-virus-infected patient revealed upregulated T_H_17 cell cytokines that became downregulated by combined treatment with pegylated IFN and ribavirin	[131]
Simian immunodeficiency virus (SIV)/HIV	Pathogenic	Induction of TGF-*β* and IL-18 during the acute phase in SIV-infected rhesus macaques proposed to be associated with induction of IL-17-producing NKT cells	[132]
	Protective	Association of disease progression with loss of T_H_17 and induction of T_Reg_ cells; T_H_17 cell frequency correlated negatively with viral load	[63, 133, 134]
Herpes simplex virus (HSV-1)		Infiltration of T_H_1 preceded T_H_17 cells, the latter showed lower responsiveness ability to HSV-1; diminished stromal keratitis severity in IL-17R^−/−^-infected mice and upon IL-17 neutralization in WT mice	[135]

(5) Nematode infection			

*Trichinella spiralis *	Pathogenic	Correlation of T_H_17 response with increase of smooth muscle contraction probably causing gut dysfunction; association of IL-17/IL-23 axis induction with increased mortality in mice coinfected with malaria and nematode	[136]
